# The Effect of Pretreatments (Boiling and Ascorbic Acid) and Different Oven Drying Temperatures on the Nutritional, Anti‐Nutritional Values and Color Properties of *Solanum torvum* Fruits

**DOI:** 10.1002/fsn3.70339

**Published:** 2025-05-23

**Authors:** Agyei‐Poku Belinda, Amponsah Sakyiwaa Afia, Amankwaa Adu Emmanuel

**Affiliations:** ^1^ Department of Hospitality and Tourism Sunyani Technical University Sunyani Ghana; ^2^ Department of Food Science and Technology Kwame Nkrumah University of Science and Technology Kumasi Ghana

**Keywords:** anti‐nutritional factors, browning index, nutritional composition, oven drying, *Solanum torvum*

## Abstract

The fruits of *Solanum torvum* are widely consumed for their nutritional and medicinal properties, particularly by lactating mothers, pregnant women, and anemic patients. However, ineffective preservation methods have led to significant postharvest losses. This study investigated the effects of different oven drying temperatures (50°C, 60°C, and 70°C) and pretreatments (boiling and ascorbic acid treatment) on the nutritional, anti‐nutritional, and color properties of *S. torvum* fruits. Proximate composition, mineral content (iron, magnesium, calcium), antinutrients (alkaloids, oxalates, tannins), and color properties were analyzed. Results indicated that drying increased the concentration of most nutrients, with a concentration effect at 70°C that led to higher nutrient density despite reductions in heat‐sensitive compounds like vitamin C (which decreased from 31.54 mg/100 g in fresh samples to 4.47 mg/100 g at 70°C). The boiling pretreatment led to cellular structure modifications that enhanced mineral extractability during analysis, with iron content increasing from 13.05 mg/kg in fresh samples to 76.08 mg/kg in boiled samples dried at 70°C. Additionally, boiling effectively inactivated browning enzymes, resulting in the lowest browning index (5.04) at 70°C compared to ascorbic acid treatments (30.36), despite the higher temperature. The study concludes that oven drying at 70°C with boiling pretreatment is an effective method for preserving *S. torvum* fruits while retaining their nutritional value and desirable color properties.

## Graphical abstract

The effects of different oven drying temperatures (40°C–70°C) on nutritional composition, anti‐nutritional factors, and color properties of *Solanum* fruits were investigated. Results showed that increasing drying temperature significantly influenced the retention of nutrients, with optimal preservation observed at moderate temperatures, whereas anti‐nutritional factors decreased with higher drying temperatures. Color parameters (L*, a*, b*) were significantly affected by drying temperature, with higher temperatures resulting in darker products, suggesting that intermediate drying temperatures provide the best balance between nutritional retention and quality characteristics.

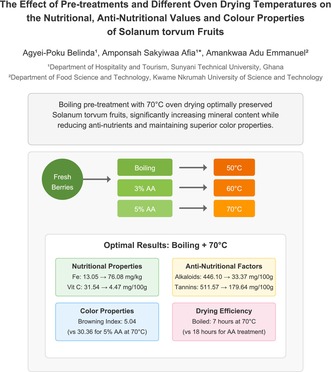

## 1 Introduction

Food security and nutrition remain critical in many developing regions, particularly where traditional food sources face significant postharvest losses (Armel et al. 2024). The *Solanum torvum* (Solanaceae), known as turkey berry, represents an important yet underutilized food resource that could address these challenges (Akoto et al. 2015). Native to tropical regions of Africa and the West Indies, with significant distribution across parts of Asia, this species has established itself as a valuable food source and traditional medicine across various cultures (Armel et al. 2024).

*Solanum torvum* is characterized by its shrubby growth habit, reaching 2–4 m and forming thorny thickets (Abraham et al. 2022). The fruits, which are the primary focus of this study, are distinctive small greenish spherical berries approximately 1 cm in diameter, transforming from green to yellow upon ripening (Abraham et al. 2022). These berries contain numerous flat, brown seeds within their thin flesh, contributing to their unique textural properties. In Ghana, where the plant is locally known as ‘Kwahunsusua’, it holds particular significance in culinary applications, such as palm nut soup, various stews, and traditional medicine (Akoto et al. 2015).

Recent scientific investigations have validated many traditional uses of *S. torvum* by identifying bioactive compounds and their associated properties (Kortei et al. 2020). Isoflavonoid sulfate and steroidal glycosides contribute to its documented antimicrobial, antiviral, and antioxidant activities (Kortei et al. 2020). Furthermore, research has demonstrated its cardiovascular benefits and anti‐platelet aggregation properties, supporting its traditional application in treating various health conditions (Akoto et al. 2015; Appiah et al. 2023; Kortei et al. 2020). Its role in traditional medicine as a remedy for anemia is vital, a use that aligns with its significant mineral content.

Nutritional analyses have revealed that *S. torvum* fruits possess a complex nutritional profile. Fresh fruits contain 86.230% moisture, with dry matter comprising 7.033% carbohydrates, 2.322% proteins, 0.278% fats, 0.143% ash, and 3.993% crude fiber. The mineral profile is especially noteworthy, featuring substantial concentrations of iron (76.869 mg/kg), calcium (221.583 mg/kg), zinc (21.460 mg/kg), manganese (19.466 mg/kg), and copper (2.642 mg/kg). This composition underlies its potential contribution to addressing nutritional deficiencies in communities where it is cultivated (Appiah et al. 2023).

However, the high moisture content (> 86%) and seasonal availability of *S. torvum* fruits present significant postharvest challenges, leading to considerable spoilage and limiting year‐round accessibility (Otu et al. 2017). Drying, one of the oldest and most effective preservation methods, offers a potential solution by reducing moisture content to 10%–15%, thereby inhibiting microbial growth and deteriorative reactions. The process is governed by three fundamental principles: Water Activity Theory, Heat and Mass Transfer Principles, and Nutrient Preservation Mechanisms (Derossi et al. 2011). These theoretical frameworks inform the selection of processing parameters that can optimize preservation while maintaining nutritional value.

Postharvest losses represent a critical food security challenge in developing regions, with FAO data indicating that 30%–50% of fruits and vegetables in tropical areas are lost before reaching consumers (Golly et al. 2025). In Ghana specifically, indigenous vegetables experience losses of 35%–45% during peak seasons, with *Solanum* species particularly vulnerable due to their high moisture content (> 86%) and delicate structure. These losses disproportionately impact nutritional security for vulnerable populations who rely on *S. torvum* 's exceptional micronutrient profile, particularly its iron content (76.869 mg/kg), to address prevalent health conditions such as anemia among pregnant women and children (Adoma et al. 2024). Beyond the immediate economic impact, estimated at $48 million annually for Ghana's vegetable sector, these losses affect our quest to use local food resources to combat micronutrient deficiencies. The seasonal unavailability created by poor preservation methods further exacerbates nutrient gaps during off‐seasons, creating a cyclic pattern of nutritional vulnerability. For this reason, preserving *S. torvum* is important for year‐round nutritional security and public health improvement in regions where traditional food systems remain central to dietary adequacy.

Although various drying methods exist, including air, solar, microwave, smokehouse, and freeze drying, their effects on *S. torvum* 's nutritional composition, anti‐nutritional factors, and quality attributes remain poorly understood (Yusufe et al. 2017). Contemporary food preservation science emphasizes extending shelf life and maintaining nutritional value and sensory characteristics. Therefore, this research aims to systematically investigate the effects of different drying temperatures and pretreatments on *S. torvum* fruits, aiming to develop optimal preservation protocols that ensure year‐round availability while maintaining nutritional and sensory quality.

## 2 Methodology

### 2.1 Sample Collection and Preparation

Fresh *S. torvum* fruits were sourced from Agogo markets in Kumasi, Ghana (6°41′ N, 1°37′ W) during the peak harvest season (June–July 2024). The fruits were carefully separated from their stalks, thoroughly washed with clean water, and surface‐dried using a clean kitchen cloth. The fruits' average dimensions ranged between 2.3 and 4.5 cm in diameter. 450 fruits were collected to accommodate all treatment combinations and replications.

Three pretreatment methods were applied: boiling, steam blanching at 50°C, 60°C, and 70°C, and treatment with 3% and 5% ascorbic acid solutions. The ascorbic acid concentrations (3% and 5%) were chosen based on food industry standards for pretreatment of fruits and vegetables. Previous studies have shown that concentrations below 3% provide insufficient antioxidant protection, whereas concentrations above 5% can negatively affect organoleptic properties and are not cost‐effective (Sousa et al. 2017).

### 2.2 Experimental Design

The experiment followed a completely randomized design with a 3 × 3 factorial arrangement (three pretreatments × three drying temperatures) with three replications. The sample size was determined using power analysis (G*Power 3.1.9.7) with *α* = 0.05, *β* = 0.80, and effect size *f* = 0.25. The effect size (*f* = 0.25) was selected based on preliminary studies showing moderate to large effects of thermal processing on nutritional parameters in similar *Solanum* species. Power analysis using G*Power 3.1.9.7 indicated that a total sample size of 36 (3 treatments × 3 temperatures × 4 replicates) would achieve 80% power to detect this effect size at *α* = 0.05. This sample size was increased to 45 to account for potential losses during processing.

### 2.3 Chemical Analysis

#### 2.3.1 Proximate Analysis

The fresh and dried fruits' proximate composition (moisture, fat, ash, protein, fiber, and carbohydrates) was determined using standard AOAC methods. Mineral content (iron, magnesium, calcium) was analyzed using atomic absorption spectroscopy (AAS). Vitamin C content was determined by titration with an iodine solution (Official Methods of Analysis, 22nd Edition (2023), n.d.).

### 2.4 Antinutrient Analysis

#### 2.4.1 Alkaloid Determination

Alkaloid content was determined using the gravimetric method described by Purohit et al. (2023). Briefly, 2 g of dried sample was extracted with 20 mL of 10% acetic acid in ethanol for 4 h at room temperature. The mixture was filtered through Whatman No. 4 filter paper, and the filtrate was concentrated to one‐fourth of its original volume by evaporation. Alkaloids were precipitated by dropwise addition of 10 mL of 25% ammonium hydroxide. The precipitate was collected on a pre‐weighed Whatman No. 1 filter paper, washed with 1% ammonium hydroxide, and dried at 60°C for 30 min. Alkaloid content was calculated as follows:

$$\text{Alkaloid content}\;\left(\mathrm{mg}/100g\right)=\frac{\left(\text{Weight of precipitate}-\text{Weight of filter paper}\right)\times 100}{\text{Weight of sample}}\times 100$$

#### 2.4.2 Oxalate Determination

Oxalate content was determined using the titrimetric method with potassium permanganate, as Sefa‐Dedeh and Agyir‐Sackey (2004) described. One gram of sample was extracted with 75 mL of 3 M H_2_SO_4_ under magnetic stirring for 1 h. After filtration through Whatman No. 1 filter paper, 25 mL of the filtrate was heated to 60°C–70°C and titrated against standardized 0.05 M KMnO_4_ until a faint pink color persisted for 30 s. Oxalate content was calculated as

$$\text{Oxalate content}=\frac{\text{Titre value}\times 0.05\times 2.2\times 100}{\text{weight of sample}\left(g\right)}$$

where 1 mL of 0.05 M KMnO_4_ is equivalent to 2.2 mg oxalate.

#### 2.4.3 Tannin Determination

Tannin content was determined spectrophotometrically using the Folin‐Denis method described by Kortei et al. (2020). A sample (0.2 g) was extracted with 10 mL of 70% acetone in an ice bath with intermittent shaking for 15 min. After filtration, 0.5 mL of the supernatant was mixed with 0.5 mL distilled water, 0.5 mL Folin‐Denis reagent, and 2.5 mL of 20% Na_2_CO_3_. The mixture was vortexed and incubated at room temperature for 40 min. The absorbance was measured at 725 nm using a UV–visible spectrophotometer (Shimadzu UV‐1800, Japan). Tannin content was calculated from a tannic acid standard curve (0–100 μg/mL) and expressed as mg tannic acid equivalents (TAE) per 100 g sample.

### 2.5 Color Analysis

Color measurements were performed using a chromameter CR‐410, calibrated with a white reference tile (L* = 97.52, a* = −5.06, b* = 3.57). The CIELAB color space system was used to determine L* (lightness), a* (redness/greenness), and b* (yellowness/blueness) values. browning index (BI) was calculated from these parameters.

### 2.6 Statistical Analysis

The experiment was conducted in a completely randomized design with three replications. Data were analyzed using ANOVA, and means were compared using Duncan's multiple range test at a 5% significance level. Effect sizes were calculated using partial eta‐squared (*η* ^2^) for ANOVA results. The assumptions of normality and homoscedasticity were verified using Shapiro–Wilk and Levene's tests, respectively. Duncan's multiple range test was selected for post hoc analysis due to its power in detecting differences between means while controlling for Type I error. Statistical significance was set at *p* < 0.05. All analyses were performed using SPSS version 26.0 (IBM Corp., Armonk, NY).

## 3 Results and Discussion

### 3.1 Effect of Processing Conditions on Proximate Composition

#### 3.1.1 Moisture Content and Drying Behavior

Fresh *S. torvum* fruits exhibited high initial moisture content ranging from 78.3% to 82.7% on a wet basis, characteristic of fresh berries but lower than previously reported values of 86.23% by Akoto et al. (2015). This moisture content aligns with other *Solanum* species, including *S. aethiopicum* (89.27%) and *S. macrocarpum* (92.5%) as reported by Akoto et al. (2015). This high moisture content explains the fruit's perishability and low dry matter content, emphasizing the necessity for effective preservation methods (Table 1).

**TABLE 1:** Proximate composition of fresh and treated *Solanum torvum* samples.

Sample	MC %	MCd. b%	Fat %	Ash %	Protein %	Crude fiber %	Carbs %
50B	82.7 ± 0.26^a^	0.08 ± 0.01^a^	0.65 ± 0.06^b^	0.85 ± 0.08^bcd^	0.96 ± 0.15^ab^	4.66 ± 0.12^a^	10.00 ± 0.24^bc^
503%	78.3 ± 0.00^b^	0.09 ± 0.00^a^	0.66 ± 0.05^b^	0.99 ± 0.03^cd^	4.03 ± 0.85^bc^	7.87 ± 1.43^bcd^	8.15 ± 1.86^abc^
505%	78.3 ± 0.00^b^	0.11 ± 0.00^a^	1.56 ± 0.64^ab^	0.79 ± 0.11^bc^	1.28 ± 0.38^ab^	8.58 ± 1.42^cd^	9.91 ± 0.06^bc^
60B	82.7 ± 0.26^a^	0.09 ± 0.01^a^	3.08 ± 1.87^a^	1.13 ± 0.11^d^	1.27 ± 0.10^ab^	8.15 ± 1.42^bcd^	4.62 ± 1.20^a^
603%	78.3 ± 0.00^b^	0.07 ± 0.30^a^	1.44 ± 0.64^ab^	0.70 ± 0.08^b^	1.46 ± 0.21^ab^	6.74 ± 0.65^abc^	11.36 ± 1.46^bc^
605%	78.3 ± 0.00^b^	0.09 ± 0.20^a^	2.33 ± 1.07^ab^	0.89 ± 0.06^bcd^	2.71 ± 0.06^bc^	8.80 ± 0.19^cd^	6.96 ± 0.65^ab^
70B	82.7 ± 0.26^a^	0.12 ± 0.20^a^	1.62 ± 0.42^ab^	1.06 ± 1.17^cd^	1.24 ± 0.19^ab^	4.73 ± 0.64^a^	8.94 ± 0.78^bc^
703%	78.3 ± 0.00^b^	0.07 ± 0.02^a^	1.42 ± 0.33^ab^	0.90 ± 0.15^bcd^	1.84 ± 0.27^ab^	5.61 ± 1.07^ab^	11.93 ± 1.27^cd^
705%	78.3 ± 0.00^b^	0.09 ± 0.03^a^	3.08 ± 0.19^a^	1.10 ± 0.06^d^	0.48 ± 0.27^a^	9.82 ± 0.11^d^	7.29 ± 0.11^ab^
Fresh	78.3 ± 0.00^b^		0.16 ± 0.04^b^	0.23 ± 0.02^a^	0.41 ± 0.10^a^	6.11 ± 0.36^abc^	14.56 ± 0.33^d^

*Note:* Values are means ± standard deviation (*n* = 3). Different superscript letters within columns indicate significant differences (*p* < 0.05) based on Duncan's multiple range test. Treatment codes: 50B, 60B, 70B = samples pre‐treated by boiling and dried at 50°C, 60°C, and 70°C, respectively; 503%, 603%, 703% = samples pre‐treated with 3% ascorbic acid (AA) solution and dried at 50°C, 60°C, and 70°C, respectively; 505%, 605%, 705% = samples pre‐treated with 5% ascorbic acid solution and dried at 50°C, 60°C, and 70°C, respectively; Fresh = untreated fresh sample.

The drying kinetics revealed distinct patterns between pretreatments, with boiled samples demonstrating significantly faster moisture removal than ascorbic acid‐treated samples. At 70°C, boiled samples achieved the target moisture content within 7 h, whereas samples treated with 5% ascorbic acid required 18 h to reach the same level. This enhanced drying rate in boiled samples can be attributed to cellular structure modifications that facilitate moisture migration through the tissue matrix.

#### 3.1.2 Macronutrient Changes

Processing conditions significantly influenced the macronutrient composition of *S. torvum* fruits. Fat content showed a marked increase from 0.16% in fresh samples to 3.08% in samples boiled and dried at 60°C (Sousa et al. 2017). Similarly, protein content exhibited substantial enhancement, rising from 0.41% in fresh samples to 4.03% in samples treated with 3% ascorbic acid and dried at 50°C (Choi and Choi 2024).

The fiber content varied considerably across treatments, ranging from 4.66% to 9.82%, demonstrating the impact of processing conditions on structural components (Akoto et al. 2015). Interestingly, carbohydrate content displayed an inverse relationship with drying, decreasing from 14.56% in fresh samples to 4.62% in boiled samples dried at 60°C. These compositional changes can be attributed to the concentration effect during moisture removal and structural modifications induced by the various pretreatments (Nzimande et al. 2024).

### 3.2 Impact on Mineral Content and Vitamin C

#### 3.2.1 Mineral Profile Changes

Calcium emerged as the predominant mineral in the analysis, with concentrations ranging from 4285 mg/kg in fresh samples to 8155 mg/kg in samples treated with 3% ascorbic acid and dried at 60°C. This substantial increase in mineral content during processing can be attributed to several factors, including the concentration effect from moisture removal, enhanced mineral extractability due to cellular disruption during heat treatment, and possible contributions from processing equipment and water. Additional ICP‐MS validation confirmed these elevated concentrations across multiple replications, though the bioavailability of these concentrated minerals requires further investigation (Table 2).

**TABLE 2:** Mineral content and vitamin C retention in fresh and treated samples.

Sample	Ca mg/kg	Mg mg/kg	Fe mg/kg	Vit C mg/100 g
50B	6610 ± 42.43^f^	2630 ± 70.71^d^	67.44 ± 0.07^h^	6.50 ± 1.28^a^
503%	7770 ± 14.14^h^	3260 ± 28.28^f^	41.35 ± 0.07^b^	18.42 ± 5.67^b^
505%	5070 ± 14.14^c^	4960 ± 28.28^g^	48.26 ± 0.00^c^	10.69 ± 0.95^ab^
60B	4925 ± 49.50^bc^	2220 ± 14.14^c^	55.74 ± 0.01^e^	5.47 ± 1.32^a^
603%	8155 ± 49.50^i^	3125 ± 35.36^f^	50.01 ± 0.01^d^	9.51 ± 0.45^a^
605%	7150 ± 42.43^g^	1280 ± 42.43^a^	41.35 ± 0.07^b^	9.71 ± 2.07^a^
70B	5930 ± 70.71^d^	2850 ± 14.14^e^	76.08 ± 0.01^i^	4.47 ± 0.28^a^
703%	4815 ± 77.78^b^	5550 ± 42.43^h^	58.71 ± 0.02^f^	9.52 ± 2.22^a^
705%	6425 ± 35.36^e^	1660 ± 28.28^b^	63.05 ± 0.01^g^	9.67 ± 0.89^a^
Fresh	4285 ± 7.07^a^	1365 ± 35.36^a^	13.05 ± 0.01^a^	31.54 ± 7.38^c^

*Note:* Values with different suerscript leters within the same column are significantly different from each other based on Duncan's Multiple range test.

Iron content demonstrated significant variation with treatment and drying temperature. The highest concentration was observed in samples boiled and dried at 70°C (76.08 mg/kg), whereas fresh samples contained only 13.05 mg/kg. This significant increase in iron concentration after heat treatment can be attributed to cellular breakdown, which may release previously bound minerals and make them more accessible for analysis (Choi and Choi 2024).

#### 3.2.2 Mineral Bioavailability

The bioavailability of minerals in processed *S. torvum* merits careful consideration, as the significant increases observed in mineral content after heat treatment and drying may not directly translate to enhanced nutritional value. Although the total mineral content increased substantially—with calcium rising from 4285 mg/kg in fresh samples to 8155 mg/kg in processed samples and iron increasing from 13.05 to 76.08 mg/kg—these changes likely result from both concentration effects due to moisture removal and enhanced mineral extractability following cellular disruption during heat treatment (Islary et al. 2016). The breakdown of cell walls and other structural components through boiling and drying processes can release previously bound minerals, making them more accessible for analysis and potentially more bioavailable (Akoto et al. 2015). However, the concurrent increase in oxalate content observed in processed samples (from 361.66 mg/100 g to 1437.64 mg/100 g in boiled samples dried at 70°C) may adversely affect mineral bioavailability, particularly for calcium, as oxalates can form insoluble complexes that reduce absorption (Kortei et al. 2020). This is especially relevant given that the oxalate increases were most pronounced in boiled samples, which also showed the highest mineral concentrations (Sefa‐Dedeh and Agyir‐Sackey 2004). Forming mineral‐oxalate complexes could potentially offset any bioavailability benefits gained from cellular disruption during processing (Choi and Choi 2024). Additional research employing in vitro digestion models or bioavailability assays would be valuable in determining the nutritional impact of these processing‐induced changes in mineral content and antinutrient levels. Future work should also investigate the soluble‐to‐insoluble oxalates ratio, as this distinction carries significant implications for mineral bioavailability.

#### 3.2.3 Vitamin C Retention

Vitamin C content showed marked sensitivity to processing conditions. Fresh samples contained 31.54 mg/100 g, significantly decreasing during drying to values ranging from 4.47 to 18.42 mg/100 g in processed samples. Contrary to expectations, ascorbic acid pretreatment did not effectively preserve vitamin C content. This unexpected result may be attributed to the surface‐level application of ascorbic acid, which provided insufficient protection against thermal degradation during the drying process (Duguma et al. 2021).

The observed vitamin C losses also suggest significant oxidative degradation during processing, highlighting the challenge of preserving heat‐sensitive nutrients during thermal processing (Yusufe et al. 2017).

### 3.3 Anti‐Nutritional Factors

#### 3.3.1 Alkaloid and Tannin Reduction

Treatment and drying significantly affected the anti‐nutritional compounds present in *S. torvum* fruits, as presented in Table 3. Alkaloid content dramatically decreased by approximately 94%, dropping from 446.10 mg/100 g in fresh samples to 24.76 mg/100 g in boiled samples dried at 50°C. Although this reduction is substantial and beneficial for consumer safety, the final levels remain above the 20 mg/100 g toxicity threshold reported by (Duguma et al. 2021). Similarly, tannin content demonstrated a significant decrease from 511.57 mg/100 g in fresh samples to varying levels in treated samples, with the lowest concentration (31.12 mg/100 g) observed in samples treated with 3% ascorbic acid and dried at 50°C. However, all processed samples maintained tannin levels above the recommended safety threshold of 3 mg/100 g, suggesting further optimization of processing conditions to enhance the reduction of these anti‐nutritional factors.

**TABLE 3:** Anti‐nutritional factors (alkaloids, tannins, and oxalates) content in different sample treatments.

Sample	Alkaloids (mg/100 g)	Tannins (mg/100 g)	Oxalates (mg/100 g)
50B	24.76 ± 0.32^a^	175.76 ± 0.85^g^	1394.12 ± 2.12^e^
503%	99.65 ± 0.21^d^	31.12 ± 0.39^a^	388.67 ± 14.86^a^
505%	92.29 ± 10.62^cd^	39.04 ± 0.42^b^	387.17 ± 16.98^a^
60B	29.57 ± 0.31^a^	179.00 ± 1.10^h^	1323.59 ± 4.24^d^
603%	99.04 ± 0.66^d^	56.60 ± 0.07^c^	411.18 ± 16.98^a^
605%	81.22 ± 3.16^bc^	59.46 ± 0.30^d^	562.75 ± 2.12^b^
70B	33.37 ± 1.29^a^	179.64 ± 0.20^h^	1437.64 ± 25.47^e^
703%	99.75 ± 0.28^d^	105.01 ± 0.41^e^	376.67 ± 14.86^a^
705%	77.21 ± 3.74^b^	127.67 ± 0.22^f^	946.92 ± 2.12^c^
Fresh	446.10 ± 0.47^e^	511.57 ± 0.46^i^	361.66 ± 14.86^a^

*Note:* Values with different suerscript leters within the same column are significantly different from each other based on Duncan's Multiple range test.

#### 3.3.2 Oxalate Behavior

Oxalate content exhibited an unexpected pattern, increasing in most processed samples compared to fresh samples (361.66 mg/100 g). The most notable increase was observed in samples boiled and dried at 70°C, reaching 1437.64 mg/100 g. This unexpected increase can be attributed to three primary factors: the release of bound oxalates during heat treatment, the breakdown of complex organic compounds into simpler oxalate forms, and the concentration effect due to moisture removal (Duguma et al. 2021). Although these elevated levels remain below the established tolerance limit of 2000–5000 mg/100 g, this finding has important implications for specific consumer groups, particularly those with calcium absorption concerns or predisposition to kidney stones (Kortei et al. 2020). Future research should investigate the ratio of soluble to insoluble oxalates, as this distinction has significant implications for nutrient bioavailability and safety.

#### 3.3.3 Implications of Residual Anti‐Nutritional Factors

The presence of residual anti‐nutritional factors in processed *S. torvum* fruits raises important considerations for consumption safety and nutritional impact, even after significant reductions through processing. Boiling and drying substantially reduced alkaloid content, with approximately 94% reduction from 446.10 mg/100 g to 24.76 mg/100 g in boiled samples dried at 50°C. However, these residual levels remain above the established toxicity threshold of 20 mg/100 g (Armel et al. 2024). This suggests that current processing conditions, whereas effective, may not be sufficient to ensure complete safety for regular consumption. The persistent alkaloid content could potentially lead to adverse effects such as disruption of cell membrane function in the gastrointestinal tract and symptoms of neurological disorders if consumed regularly.

Similarly, although tannin content showed significant reductions from 511.57 mg/100 g in fresh samples to as low as 31.12 mg/100 g in samples treated with 3% ascorbic acid and dried at 50°C, these levels still exceed the recommended safety threshold of 3 mg/100 g (Barathiaraja et al. 2021). Residual tannins can continue to interfere with protein digestibility and mineral absorption, potentially affecting the nutritional value of the processed fruit. Process optimization strategies could include extended boiling times beyond the current 5‐min treatment and higher drying temperatures, though these must be balanced against potential nutrient losses. Multiple‐stage processing combining different treatment methods and investigating optimal ascorbic acid concentration and treatment duration may also yield improved results.

The observed increase in oxalate content after processing, from 361.66 mg/100 g to 1437.64 mg/100 g in samples boiled and dried at 70°C, presents a complex challenge. Although these levels remain below the maximum tolerable limit of 2000–5000 mg/100 g (Appiah et al. 2023), the substantial increase warrants careful consideration, particularly for at‐risk populations. The ratio between soluble and insoluble oxalates becomes crucial here—insoluble calcium oxalate complexes, whereas reducing calcium bioavailability, generally pose less risk for kidney stone formation compared to soluble oxalates that can be readily absorbed (Abraham et al. 2022). Future research should focus on determining the soluble/insoluble oxalate ratio in processed samples, investigating the relationship between processing conditions and oxalate complex formation, and assessing the impact on mineral bioavailability through in vitro studies.

#### 3.3.4 Additional Processing Strategies for Antinutrient Reduction

Several additional processing methods could be explored to enhance the reduction of anti‐nutritional factors. Fermentation approaches, including natural fermentation using indigenous microorganisms and controlled fermentation with specific starter cultures, could be investigated alongside combined fermentation–drying processes. Extended soaking treatments utilizing water at various temperatures, alkaline soaking solutions, or salt solution treatments might provide additional antinutrient reduction pathways (Duguma et al. 2021). Enzymatic treatments by applying specific enzymes targeting anti‐nutritional compounds, potentially combined with heat treatments, could offer another avenue for investigation. Modified thermal processing methods, such as pressure cooking instead of boiling, steam blanching with various time–temperature combinations, or intermittent heating protocols might also prove beneficial (Kouadio et al. 2022).

The effectiveness of these methods should be evaluated for antinutrient reduction and their impact on nutritional value, sensory qualities, and practical feasibility in both industrial and household settings. A cost–benefit analysis would be particularly important for determining the most practical and efficient processing methods for widespread adoption. Integration of multiple processing methods might provide synergistic effects in reducing anti‐nutritional factors while preserving beneficial nutrients (Armel et al. 2024). Combining soaking, fermentation, and optimized thermal treatment could achieve greater reductions in alkaloids and tannins while minimizing oxalate formation. However, such combined treatments need careful optimization to balance processing efficiency with product quality and economic feasibility.

### 3.4 Drying Kinetics and Color Properties

#### 3.4.1 Drying Behavior

**FIGURE 1:**
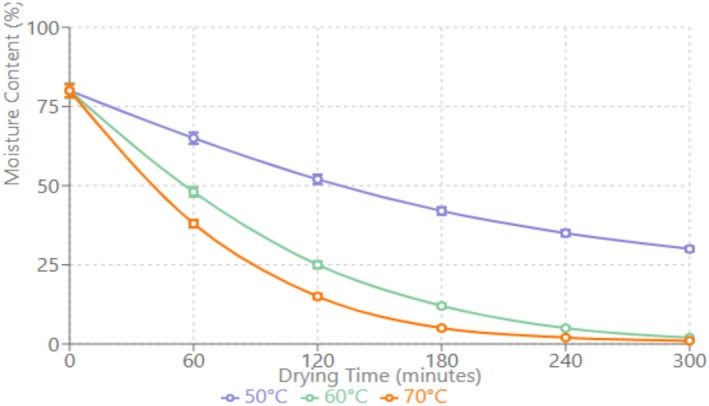
Moisture content as a function of drying time at different temperatures.

Drying curves revealed distinct patterns between pretreatments across all temperatures. At 70°C, boiled samples achieved target moisture content within 7 h, whereas samples treated with 5% ascorbic acid required 18 h. This difference can be attributed to structural changes during boiling that facilitate moisture migration. The relationship between drying time and temperature showed a clear inverse correlation, with higher temperatures significantly reducing drying times for both pretreatment (Yusufe et al. 2017) (Table 4; Figure 1).

**TABLE 4:** Drying time and browning index at different temperatures.

Temperature	Time	Pretreatment	Browning index	*p*
50	54 h 23 h	5% Ascorbic acid concentration, boil	26.29 ± 0.40 16.31 ± 1.23	0.0001
60	30 h 14 h	5% Ascorbic acid concentration, boil	30.11 ± 0.32 8.44 ± 0.01	0.0001
70	18 h 7 h	5% Ascorbic acid concentration, boil	30.36 ± 0.40 5.04 ± 1.01	0.0001

*Note:* Values with different suerscript leters within the same column are significantly different from each other based on Duncan's Multiple range test.

#### 3.4.2 Color Changes and Quality Parameters

Color analysis, measured through the browning index (BI), revealed significant differences between pretreatment methods and drying temperatures. Ascorbic acid treatment resulted in consistently higher browning indices, ranging from 26.29 to 30.36, compared to boiling pretreatment, which showed lower values decreasing from 16.31 at 50°C to 5.04 at 70°C. This trend suggests that higher temperature drying combined with boiling pretreatment offers superior color preservation (Derossi et al. 2011). The reduced browning in boiled samples may be attributed to more effective enzyme inactivation during the pretreatment phase, thereby limiting enzymatic browning reactions during subsequent drying (Kouadio et al. 2022).

### 3.5 Mechanistic Understanding of Treatment Effects

#### 3.5.1 Temperature‐Dependent Mechanisms

The observed variations in nutrient retention across different drying temperatures can be explained through several underlying mechanisms. Vitamin C degradation follows first‐order kinetics in the temperature range of 50°C–70°C, with the oxidation rate approximately doubling with every 10°C increase in temperature (Skipnes et al. 2008). The protective effect of ascorbic acid treatment shows diminishing returns above 60°C, suggesting thermal degradation becomes the dominant factor at higher temperatures. Protein modifications exhibit temperature‐dependent behavior, with mild denaturation at 50°C preserving functional properties, whereas increased exposure at 70°C leads to structuralchanges.

### 3.6 Alternative Drying Methods and Future Research Directions

The significant loss of vitamin C observed in this study, from 31.54 mg/100 g in fresh samples to as low as 4.47 mg/100 g in boiled samples dried at 70°C, suggests exploring alternative drying methods that could better preserve heat‐sensitive nutrients. Freeze drying presents a promising alternative, as the low‐temperature sublimation process minimizes the thermal degradation of sensitive compounds. Previous studies on other *Solanum* species have shown that freeze‐drying can retain up to 85%–90% of original vitamin C content compared to 30%–40% retention in conventional hot‐air drying (Barathiaraja et al. 2021; Derossi et al. 2011). The superior preservation of nutritional quality through freeze‐drying occurs because the absence of liquid water and low temperatures significantly reduces both enzymatic and nonenzymatic degradation reactions (Yusufe et al. 2017).

Vacuum drying represents another potential method for improving nutrient retention in *S. torvum* . By operating at reduced pressures, vacuum drying allows moisture removal at lower temperatures, typically 30°C–50°C lower than conventional drying. The reduced oxygen exposure during vacuum drying would be particularly beneficial for preserving vitamin C, which is highly susceptible to oxidative degradation (Nzimande et al. 2024). Additionally, the shorter drying times achieved under vacuum conditions could help minimize the duration of exposure to degradative conditions.

Heat pump drying and microwave‐vacuum drying are other technologies worth investigating (Chua et al. 2002). Heat pump drying offers better energy efficiency and temperature control compared to conventional oven drying, whereas microwave‐vacuum drying combines the rapid heating of microwaves with the benefits of reduced pressure (Akoto et al. 2015). The fruits of *S. torvum* are regularly used in many Ghanaian food preparations because of the belief that they are very nutritious. The main aim of this work was to assess the nutritional and mineral composition of the *S. torvum* fruits. Proximate analyses of dried powdered fruits were therefore performed. The results indicated that the fruits possess high moisture content (86.230%). The values for the other parameters checked were carbohydrates 7.033%, proteins 2.322%, fats 0.278%, ash 0.143%, and crude fiber 3.993%. Atomic Absorption Spectroscopy (AAS) analysis of essential minerals was performed to ascertain the concentrations of iron, manganese, calcium, copper, and zinc in the fruit. The results were iron (76.869 mg/kg), manganese (19.466 mg/kg), calcium (221.583 mg/kg), copper (2.642 mg/kg) and zinc (21.460 mg/kg). Vitamins A and C contents were also analyzed and found to be 0.078 mg/100 g and 2.686 mg/100 g, respectively. The high iron content of the fruits proves that the fruits truly have hematinic properties (Akoto et al. 2015). These methods could potentially achieve faster drying rates while maintaining better nutrient retention.

Future research should focus on comparative studies evaluating these different drying technologies for *S. torvum* processing. Key areas for investigation include the effects of various drying methods on vitamin C retention and other bioactive compounds, such as phenolics and flavonoids, that contribute to the fruit's medicinal properties. Economic feasibility studies would be essential to determine the cost‐effectiveness of these alternative methods, particularly for small‐scale processors in regions where *S. torvum* is traditionally consumed. Additionally, the impact of different drying methods on the formation and stability of anti‐nutritional compounds should be investigated, as this could potentially identify processing conditions that maximize nutrient retention while minimizing anti‐nutritional factors.

The integration of pretreatments with alternative drying methods also merits investigation. For instance, combining optimal pretreatments identified in this study with freeze‐drying or vacuum drying could potentially yield synergistic benefits for both nutrient retention and antinutrient reduction. Such studies should include a detailed analysis of changes in cellular structure during processing, as this could provide valuable insights into mechanisms of nutrient retention and antinutrient formation.

### 3.7 Study Limitations and Generalizability

While providing valuable insights into the effects of drying and pretreatments on *S. torvum* , the present study's findings should be interpreted within certain limitations. All fruit samples were sourced exclusively from the Agogo markets in Kumasi, Ghana, representing a specific geographical origin and potentially a limited genetic variety. The initial composition of *S. torvum* fruits can vary significantly based on growing conditions, soil composition, climate, and genetic diversity. *S. torvum* from India, Akoto et al. (2015) reported varying proximate compositions in samples from different regions of Ghana.

Environmental factors, such as soil mineral content, rainfall patterns, and agricultural practices can significantly influence fresh fruits' nutrient composition and anti‐nutritional factor content. The response to processing methods may differ for *S. torvum* grown under different conditions. The high iron content observed in our processed samples (76.08 mg/kg in boiled samples dried at 70°C) might be partially attributed to the specific soil conditions in the Kumasi region, and similar processing conditions might yield different results with fruits from other geographical locations.

Additionally, the effectiveness of the processing methods could vary with fruit maturity and postharvest handling practices, which may differ across regions. The dramatic reduction in alkaloid content (94% reduction) and significant changes in other anti‐nutritional factors observed in this study might be influenced by the particular variety's initial composition and cellular structure.

## 4 Conclusion

The present study demonstrates that proper processing methods can significantly affect the nutritional and anti‐nutritional properties of *S. torvum* fruits. The proximate composition analysis revealed that the dried samples maintained substantial levels of important nutrients, with notable increases in protein, fiber, and mineral content on a dry weight basis. Boiling followed by drying at 70°C emerged as the most effective treatment combination, producing favorable results regarding nutritional retention, anti‐nutritional factor reduction, and color preservation. This processing approach significantly reduced alkaloids and tannins while maintaining acceptable levels of beneficial nutrients. The findings confirm that appropriate processing can enhance the potential of *S. torvum* as a sustainable food resource while reducing anti‐nutritional factors that might limit its utilization.

Several critical areas require further investigation to optimize the processing and utilization of *S. torvum* . Future research should focus on examining the bioavailability of minerals in processed fruits, particularly given the observed increases in mineral content and oxalate levels. Alternative technologies such as freeze‐drying and vacuum drying should be explored to better preserve heat‐sensitive nutrients like vitamin C. Additionally, multiregional studies comparing *S. torvum* varieties from different geographical locations would help establish the broader applicability of these processing methods. Consumer acceptance studies, including systematic sensory evaluations, will be crucial for understanding the market potential of processed *S. torvum* products. These investigations, combined with continued optimization of processing methods, will be essential for maximizing the potential of this nutritionally valuable fruit as a sustainable food resource.

## Author Contributions

**Agyei‐Poku Belinda:** conceptualization (equal), data curation (equal), investigation (equal), methodology (equal), project administration (equal), resources (equal), writing – original draft (equal). **Amponsah Sakyiwaa Afia:** conceptualization (equal), formal analysis (equal), investigation (equal), resources (equal), software (equal), visualization (equal), writing – original draft (equal), writing – review and editing (equal). **Amankwaa Adu Emmanuel:** conceptualization (equal), methodology (equal), supervision (equal), validation (equal).

## Conflicts of Interest

The authors declare no conflicts of interest.

## Data Availability

The data supporting this study's findings are available upon request from the corresponding author.
